# Susceptibility identification for seasonal influenza A/H3N2 based on baseline blood transcriptome

**DOI:** 10.3389/fimmu.2022.1048774

**Published:** 2023-01-12

**Authors:** Jing Tang, Qiumei Xu, Kang Tang, Xiaoyan Ye, Zicheng Cao, Min Zou, Jinfeng Zeng, Xinyan Guan, Jinglin Han, Yihan Wang, Lan Yang, Yishan Lin, Kaiao Jiang, Xiaoliang Chen, Yang Zhao, Dechao Tian, Chunwei Li, Wei Shen, Xiangjun Du

**Affiliations:** ^1^ School of Public Health (Shenzhen), Shenzhen Campus of Sun Yat-sen University, Shenzhen, China; ^2^ School of Public Health (Shenzhen), Sun Yat-sen University, Guangzhou, China; ^3^ Guangzhou Eighth People’s Hospital, Guangzhou Medical University, Guangzhou, China; ^4^ Department of Otolaryngology, The First Affiliated Hospital of Sun Yat-sen University, Guangzhou, China; ^5^ School of Public Health, Shantou University, Shantou, China; ^6^ Department of Chronic Disease Control and Prevention, Shenzhen Guangming District Center for Disease Control and Prevention, Shenzhen, China; ^7^ School of Public Health, The University of Hong Kong, Pokfulam, Hong Kong SAR, China; ^8^ Palos Verdes Peninsula High School, Rancho Palos Verdes, CA, United States; ^9^ Department of Rheumatology and Immunology, Nanjing Drum Tower Hospital Clinical College of Nanjing Medical University, Nanjing, China; ^10^ Key Laboratory of Tropical Disease Control, Ministry of Education, Sun Yat-sen University, Guangzhou, China

**Keywords:** influenza A/H3N2, susceptibility, immune response, gene co-expression network, high-risk population prediction model

## Abstract

**Introduction:**

Influenza susceptibility difference is a widely existing trait that has great practical significance for the accurate prevention and control of influenza.

**Methods:**

Here, we focused on the human susceptibility to the seasonal influenza A/H3N2 of healthy adults at baseline level. Whole blood expression data for influenza A/H3N2 susceptibility from GEO were collected firstly (30 symptomatic and 19 asymptomatic). Then to explore the differences at baseline, a suite of systems biology approaches - the differential expression analysis, co-expression network analysis, and immune cell frequencies analysis were utilized.

**Results:**

We found the baseline condition, especially immune condition between symptomatic and asymptomatic, was different. Co-expression module that is positively related to asymptomatic is also related to immune cell type of naïve B cell. Function enrichment analysis showed significantly correlation with “B cell receptor signaling pathway”, “immune response−activating cell surface receptor signaling pathway” and so on. Also, modules that are positively related to symptomatic are also correlated to immune cell type of neutrophils, with function enrichment analysis showing significantly correlations with “response to bacterium”, “inflammatory response”, “cAMP−dependent protein kinase complex” and so on. Responses of symptomatic and asymptomatic hosts after virus exposure show differences on resisting the virus, with more effective frontline defense for asymptomatic hosts. A prediction model was also built based on only baseline transcription information to differentiate symptomatic and asymptomatic population with accuracy of 0.79.

**Discussion:**

The results not only improve our understanding of the immune system and influenza susceptibility, but also provide a new direction for precise and targeted prevention and therapy of influenza.

## Introduction

Influenza virus causes regular seasonal epidemics and occasional severe pandemics in humans and animals (1–3). There are about 3 to 5 million cases of severe illness, and about 290 000 to 650 000 respiratory deaths each year caused by human seasonal influenza, resulting in a heavy public health concern worldwide (4). There are different manifestations for people exposed to the same environment with influenza virus, indicating individual susceptibility differences to influenza exists (5, 6). Identifying susceptible population with higher risk of infection and thus implementing targeted surveillance would be helpful for reducing the disease burden of human influenza.

The causes of host susceptibility to influenza is a complex issue, including factors from both virus (*e.g.* virulence, exposure dose, *etc.*) (6) and host (*e.g.* exposure history (7), genetic factors (8), age (9–11), nutrition (12), and other factors) (5–7). Most of the current studies focus on underlying mechanisms for obvious susceptible population like the elderly, children, pregnant women. However, for healthy adults, there also seems existing baseline differences which invoke clearly distinct antiviral and inflammatory responses (13). Studies have shown that preexisting influenza-specific CD4^+^ (14) and CD8^+^ T cells (15) in the blood play a protective role by limiting the severity of influenza illness. What’s more, natural killer (NK) cells were significantly lower in symptomatic shedders before influenza exposure at baseline level and KLRD1 as a key predictor used to predict influenza susceptibility (16). Susceptibility studies for other respiratory viruses have also been carried out. For example, the baseline activity of interferon-stimulated genes (ISG)-mediated defenses impedes infection progression, and thus it can protect against Severe Acute Respiratory Syndrome Coronavirus 2 (SARS-CoV-2) through activating beforehand by heterologous antiviral response against another virus (17). At the same time, a study on susceptibility to Respiratory Syncytial Virus (RSV) also revealed that the volunteers with neutrophilic inflammation in the airway before RSV exposure are easier to develop symptomatic infection (18). Taken together, the baseline conditions, especially the immune conditions, are very important for preventing the invasion of virus, but the whole blueprint is not yet very clear.

The key point to direct the problem above is to capture the characteristics of host at baseline before virus infection. The human influenza challenge experiment (13, 19–22) makes an ideal design, in which healthy adults are recruited with strict inclusion and exclusion criteria, and then inoculated with the same influenza virus, and finally documented with infection symptoms and virus titers to be recognized as symptomatic or asymptomatic hosts. This type of design eliminates various confounding factors especially the virus exposure. However, conducting this kind of study is difficult due to ethical concerns. We discovered several trial data from Gene Expression Omnibus (GEO) which were originally conducted to explore biological processes after infection. It is of great value to integrate these small-scale studies to investigate the question underlying influenza susceptibility, especially from the aspects of individual immune levels. Another key point lies in the types of characteristics to outline host susceptibility. Many methods have been applied in related studies to detect the characteristics of host: the differentially expressed genes (DEGs) between different conditions were identified along with their enriched functions (17, 23). differences in the proportion of immune cells were also identified based on blood transcriptome (24–26). Also, the machine learning methods were applied to identify gene markers for susceptibility of acute respiratory viral infection, which could distinguish symptomatic response based on gene expression profiles prior to viral exposure (27). Influenza susceptibility is a complex trait and methods of systems biology should be employed. In recent years, complex diseases or traits are studied more and more from the network level (28–30), for example, gene co-expression network (31–35), protein-protein interaction (PPI) network (36–39) and transcription regulation network (40–42). These methods provide a systematic view of the core pathways or key interacting genes related to the concerned traits. Currently, there is hardly any influenza studies on susceptibility investigation conducted from the perspective of systems biology and specifically based on baseline information.

In this study, human susceptibility to the seasonal influenza A/H3N2 is systematically explored based on baseline whole blood transcriptome of healthy adults from influenza challenge trials. DEGs, co-expression modules and significant immune cells were identified and functional annotation were carried out. Systematic immune functions were identified and prediction model was built. Findings in this study could shed light on the detailed mechanisms of influenza susceptibility and provide clues for precise prevention and treatment of influenza.

## Materials and methods

### Data collection

Two microarray data sets, GSE73072 (22) and GSE61754 (21), from the Gene Expression Omnibus (GEO) were collected. Datasets GSE30550 (13) and GSE17156 (19) for influenza A/H3N2 were not used due to potential overlap with GSE73072. For the data set GSE61754 (21), only the non-vaccinated subjects were used here. All of these data sets are from human influenza challenge experiments. All volunteers provided informed consent and underwent extensive pre-enrollment health screening. The volunteers without evidence of influenza H3N2 antibodies were included. Blood samples were collected at baseline and several other time points after inoculating volunteers with influenza A/H3N2 viruses and performed microarray-based transcriptome profiling. The detailed information for the datasets used in this study is shown in **Table S1**. The individuals were defined as symptomatic (representing susceptible hosts) and asymptomatic (representing un-susceptible hosts) hosts based on influenza laboratory tests and symptom status of self-reported modified Jackson scores (19). Same phenotypic labels in the original studies were used here. The final dataset includes 49 subjects (30 symptomatic and 19 asymptomatic).

### Data preprocessing

To eliminate the impact of the experimental platforms and processing methods on the data, pretreatments were carried out for the integrated dataset from three microarray experiments. Firstly, after the array probes were annotated, all the data were combined into a single matrix, where the rows represent the genes and the columns represent the samples, and the matrix are logarithmic transformed. Only genes presented in all two data sets were retained and the final matrix includes 8478 genes from 49 samples. Secondly, the raw matrix was normalized by the method of “normalizeBetweenArrays” in R package limma with default parameters (version 3.48.3) (43–46). Finally, batch effects were removed using the method of ComBat in R package sva with the default parameters (version 3.40.0) (47).

### Identification of differentially expressed genes

DEGs between asymptomatic and symptomatic hosts at baseline level were identified using the method of RankProd (Version 3.18.0) (48–50) using raw data. Specifically, genes with percentage of false predictions (*pfp*) ≤ 0.05 were defined as DEGs.

In addition, R Package limma (version=3.50.3) (45, 46, 51) was used to screen for differentially expressed genes between baseline and the following time points. The data set of GSE73072-DEE2 was used here, which included samples’ expression at multiple time points (5h, 12h, 22h, 36h, 46h, 53h, 60h,70h, 77h, 84h, 94h, 101h, 108h, 118h, 125h, 132h, 142h, 166h). The lmFit function fits a linear model with time as a factor and the subject as a blocking variable. The genes with an adjusted P (adjust.method = “BH”) ≤ 0.05 and |log2FC| > 0 were identified as differentially expressed genes (DEGs).

### Gene ontology (GO) enrichment analysis and KEGG pathway enrichment analysis

R package ClusterProfiler (version= 4.0.5) (52) was used to conduct gene ontology (GO) analysis and KEGG pathway enrichment analysis for DEGs and modules. All the genes in the gene expression profile (totally 8478 genes) were used as the background. The overrepresentation terms were determined with adjust *p* value less than 0.05 (Benjamini-Hochberg method). And minimum of 5 genes per ontology was chosen.

### Co-expression network analysis

After integrating and preprocessing the data, an 8478×49 (gene×subjects) expression profile was obtained. The variance of the expression value of each gene were calculated by function var of R, and sorted in descending order. And the top 20% variance genes (1696 genes) were selected for constructing co-expression network by using the R package WGCNA (31, 53). Firstly, to check whether there are outliers, samples were clustered using the method of hierarchical clustering by Hclust function in R. Secondly, to build a scale-free network, the soft thresholding power β was calculated by the function pickSoftThreshold in the R package of WGCNA. The threshold β was set at 6 with the scale independence reaching 0.90 and the average connectivity relatively high, which according to the standard of scale-free network. Thirdly, Pearson correlation was calculated and used to construct a weighted adjacency matrix with the soft thresholding power β. Then the weighted adjacency matrix was transformed into a topological overlap measure (TOM) by the function of blockwiseModules. TOM can be used to estimate the network connectivity of a gene. Lastly, average linkage hierarchical clustering was performed based on the TOM-based dissimilarity measure and the genes with the similar expression pattern were divided into modules. The other related parameters were set as following: TOMType = “unsigned”, minModuleSize = 15, reassignThreshold = 0, mergeCutHeight = 0.2, deepSplit = 4.

### Module-trait relationships analysis

To find the modules significantly correlated with the traits interested, module-trait relationships analysis was performed. To identify the modules related to influenza susceptibility, asymptomatic and symptomatic were regarded as two clinical phenotypes to calculate the correlation with module eigengenes (54). The p-value was calculated by the function “corPvalueStudent” in the R package of WGCNA. And the significance of modules was determined with p ≤ 0.1. In addition, to study the relationship between the co-expression models and basic immune condition, immune cells frequencies were also seen as clinical phenotypes to calculate the correlation with module eigengenes (35). Similarly, the p-value was calculated by the function “corPvalueStudent” in the R package of WGCNA and the significance of modules were determined with p ≤ 0.1.

### Immune cells frequencies estimation

To estimate the fraction of immune cells for the samples collected, R package CIBERSORT was used (24, 26). Frequencies for 18 types of immune cells were higher than 0 and p ≤ 0.05 in this study, although 22 types of immune cell were included in CIBERSORT. To test whether there are immune cell differences between asymptomatic and symptomatic hosts, the fraction of each immune cell type was compared using the method of *Wilcoxon rank-sum test* in R with p ≤ 0.1.

### Module-based classifier

The algorithm of random forest based on the Python package sklearn was used to build a classification model to differentiate host status for susceptibility of influenza A/H3N2 based on baseline characteristics. To prevent potential over fitting due to limited data, feature selection should be done first. Therefore, a module-based feature selection was proposed considering the biological significance represented by the co-expression modules. The philosophy behind is that genes in the module are functional related ones and be representative for the specific function. Representing genes from the significant co-expression modules were selected as follows:

1. In order to further narrow list, the intersection genes between co-expression modules and differentially expressed ones were selected.
2. For each targeted co-expression module with significant correlation to the susceptibility status, every 2 representative genes from this module were feed into the model that includes additional all the genes from other co-expression modules. Based on the model performance, the two genes with the highest accuracy were chosen as the representative genes of targeted co-expression module.
3. Repeated step 2 for 1000 times, and gene pair with mostly frequency was chosen as the representative genes of the target module to fed into the final model.
4. The representative genes for each co-expression module with significant correlation to the susceptibility status are merged as the final set of features. Parameter optimization was done using the method-GridSearchCV. And the parameter of class_weight was set as “balanced”. 5-fold cross-validation were used to evaluate the model and accuracy, precision, recall, F1 score, area under the receiver operating characteristics (ROC) curve (AUC) were calculated.

For strict validation, GSE73032 (with 22 symptomatic and 16 asymptomatic cases) was first chosen as the training set and the remaining one, GSE61754 with 8 symptomatic and 3 asymptomatic cases, as the external independent test set. At the same time, a final model was also trained on the bigger dataset that include all the two data sets used in this study.

## Results

### Data description

Microarray data from influenza challenge experiments (GSE73072 (22), GSE61754 (21)) were collected from Gene Expression Omnibus (GEO) (**Table S1**). Healthy adults from 18 to 45 years old were recruited and screened according to strict inclusion and exclusion principles (Detailed information has been reported previously (13, 19, 20, 22)). All the volunteers included were without evidence of influenza H3N2 antibodies. Blood samples were collected at baseline and several time points after infection and sequenced for quantifying whole blood gene expression. Subjects were defined as symptomatic (represent susceptible individuals) and asymptomatic (represent un-susceptible individuals) hosts based on influenza laboratory tests and symptom status by self-reported modified Jackson scores (19). Considering the heterogeneity among different influenza subtypes and as well as data volume in GEO, H3N2 subtype was chosen as the target in this study, which included 30 symptomatic and 19 asymptomatic subjects (see methods). Unified pretreatment was carried out, including filtering, standardization, batch effect removal (**Supplementary Figure 1**, see methods). Finally, a dataset with 8478 human genes and from 49 subjects (30 symptomatic and 19 asymptomatic subjects) for human influenza A/H3N2 virus was constructed for further analysis in this study.

### DEGs between asymptomatic and symptomatic hosts at baseline level with distinct biological processes

**Figure 1:**
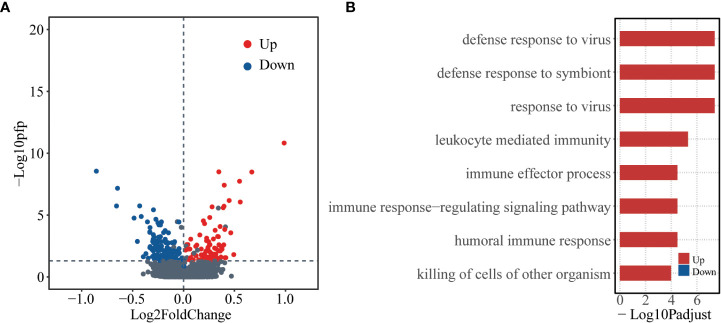
Differentially expressed genes at baseline level between asymptomatic and symptomatic hosts. **(A)** Volcano plot showing logarithmically converted fold change of gene expression between asymptomatic and symptomatic hosts on the x-axis against logarithmically converted values of *pfp* on the y-axis. The red points are up-regulated genes, and the blue points are down-regulated genes and the grey points are stable genes (asymptomatic vs symptomatic hosts). **(B)** Bar plot showing the GO biological process terms enriched for the up- (red) and down- (blue) regulated genes. The x-axis represents the logarithmically transformed *Padjust* value.

To check whether there are baseline genes associated with susceptibility of human influenza A/H3N2 infection, DEGs between asymptomatic and symptomatic hosts were identified using RankProd. A total of 223 DEGs were identified, of which, 87 were up-regulated and 136 were down-regulated (asymptomatic versus symptomatic, percentage of false predictions (*pfp*) ≤ 0.05, see methods) (**Figure 1A**).

Gene ontology (GO) enrichment analysis was further performed for up-regulated and down-regulated genes as regard with biological process (BP). The up-regulated genes tend to be enriched in immune related functions, such as, “defense response to virus”, “defense response to symbiont”, “response to virus”, *etc.* In contrast, the down-regulated genes did not significantly enrich in any functions (**Figure 1B**, **Table S2**). These results indicate that there are different bases, especially the immune basis between symptomatic and asymptomatic hosts.

### Identification of influenza susceptibility-related gene modules based on weighted co-expression networks

In the above analysis, traditional method of DEG analysis was used from the perspective of single gene. However, influenza susceptibility is a complex trait, which may not be well explained from the perspective of a single host gene or several genes. Thus, in order to explore influenza susceptibility from a systematic perspective, a co-expression network was constructed by using weighted gene co-expression network analysis (WGCNA). Genes with certain expression variation (top 20%, 1696 genes) were selected to construct the weighted co-expression network (check Methods for more details). The sample clustering tree was drawn based on the Pearson’s correlation coefficients (**Supplementary Figure 2A**). Based on the soft threshold of 6 (R^2^ ≥ 0.9) (**Supplementary Figures 2B, C**), a co-expression network was constructed with 21 modules identified (**Supplementary Figure 3A**, **Table S3**). The number of genes included in these modules ranged from 19 to 406 (**Supplementary Figure 3B**).

**Figure 2:**
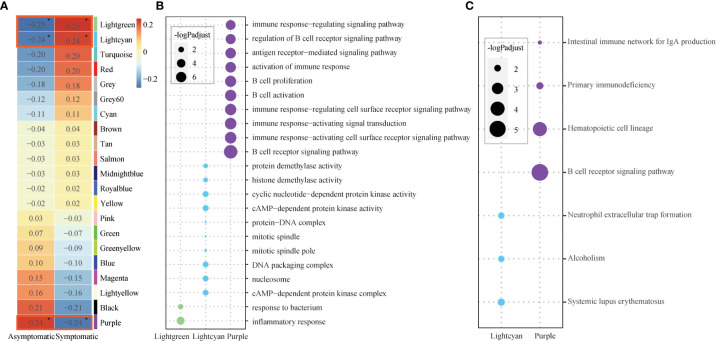
Core modules and their enriched functions. **(A)** Heatmap of Module-trait’s correlation, in which traits on the x-axis against the co-expression modules on the y-axis. The corresponding correlation was shown in the cells and color coded. The significantly related modules with the significant level were indicated by number of asterisks (* for *p* ≤ 0.1) and highlighted by red rectangles. **(B)** Bubble chart displaying the GO terms enriched for the three significant modules (Lightgreen, Lightcyan, Purple). Dot sizes are scaled to the enrichment significance. Different colors represent different modules. **(C)** Bubble chart displaying the KEGG pathways enriched for the two significant modules (Lightcyan, Purple). Dot sizes are scaled to the enrichment significance. Different colors represent different modules.

To detect the relationship between the susceptibility and modules, module-trait relationships were explored. Module Puple (*r* = 0.24, *p* = 9.7×10^-2^) was significantly positively related to asymptomatic phenotype. Modules Lightgreen (r = 0.25, *p* = 7.7×10^-2^) and Lightcyan (r = 0.25, *p* = 0.1) were significantly positively related to symptomatic phenotype (**Figure 2A**). These co-expression modules were significantly enriched in several functions, especially immune related functions, based on further functional enrichment analysis. Specifically, module Purple shows significant GO functions enrichment in immune response and B cell related functions, such as, “B cell receptor signaling pathway”, “immune response−activating cell surface receptor signaling pathway”, “immune response−activating signal transduction” and so on (**Figure 2B**, **Table S4**). Also, module Purple shows significant KEGG pathways enrichment in “B cell receptor signaling pathway” (**Figure 2C**, **Table S5**). Although the module Lightgreen is not enriched in any KEGG pathways, it is enriched in GO terms of “response to bacterium”, “inflammatory response”, (**Figures 2B, C**, **Table S4**-**5**). At the same time, the module Lightcyan is enriched in functions of “cAMP−dependent protein kinase complex”, “nucleosome” and “DNA packaging complex”, et al. (**Figure 2B**, **Table S4**) and pathways of “Neutrophil extracellular trap formation” (**Figure 2C**, **Table S5**). These results are not only consistent with the results of baseline differential expression analysis above, but also further relate the basic differences at the baseline level, especially the immune differences, to the susceptible traits.

### Different baseline immune microenvironment presents in asymptomatic and symptomatic hosts

**Figure 3:**
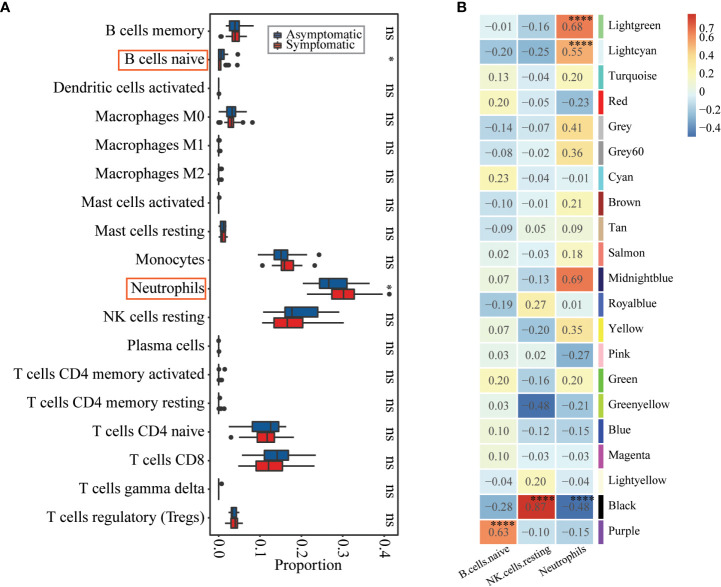
Immune cell proportions’ differences between asymptomatic and symptomatic hosts and their relationships with co-expression modules. **(A)** Boxplot showing the immune cell proportions in asymptomatic (blue) and symptomatic (red) hosts. *Wilcoxon rank-sum test* was used to determine significant differences between asymptomatic and symptomatic hosts. The significant level was indicated by number of asterisks (ns for *p >*0.1, * for *p ≤*0.1, ** for *p* ≤ 0.01, and *** for *p* ≤ 0.001) and highlighted by red rectangle. **(B)** Heatmap of Module-trait’s correlation, in which immune cell types on the x-axis against the co-expression modules on the y-axis. The corresponding correlation was shown in the cells and color coded. The significantly related modules were indicated by number of asterisks (* for *p* ≤ 0.1 and **** for *p* ≤ 0.0001) and highlighted by red rectangles.

Both of the differential expression analysis and co-expression network analysis showed that susceptibility to influenza A/H3N2 may be related to host immune status, with different immune basic environment between asymptomatic and symptomatic hosts. To test this, preexisting immune cell frequencies were estimated by CIBERSORT (24, 26). 18 of 22 types of immune cells were identified in these 49 samples (**Figure 3A**, detailed proportions in **Supplementary Figure 4**). Then the immune cell proportions were compared between the symptomatic and asymptomatic hosts. B cell naïve, neutrophils were significantly different between these two groups (**Figure 3A**, *Wilcoxon rank-sum test*, with *p-value* of 5.20×10^-2^, 6.30×10^-2^, respectively). What’s more, the proportion of B cell naïve (mean proportion of 3.00×10^-3^ and 5.66×10^-3^ in symptomatic and asymptomatic hosts) were higher in asymptomatic hosts (**Figure 3A**, **Supplementary Figure 4**). While the proportion of neutrophils (mean proportion of 2.77×10^-1^ and 2.25×10^-1^ in symptomatic and asymptomatic hosts) seem have a higher proportion in symptomatic hosts (**Figure 3A**, **Supplementary Figure 4**). There results were consistent with the above DEGs and co-expression network analysis (**Figures 1B**, **2B, C**). Additionally, the relationship between these significant three types of immune cells (naïve B cells, neutrophils) and three co-expression modules identified above were explored. Specifically, both module Lightgreen and Lightcyan were significantly positively correlated with neutrophils; module Purple were significantly positively correlated with B cell naïve (**Figure 3B**), which were consistent with the enriched functions of those modules (**Figure 2B**, **Table S4**).

### Differential response after influenza infection presents in asymptomatic and symptomatic hosts

**Figure 4:**
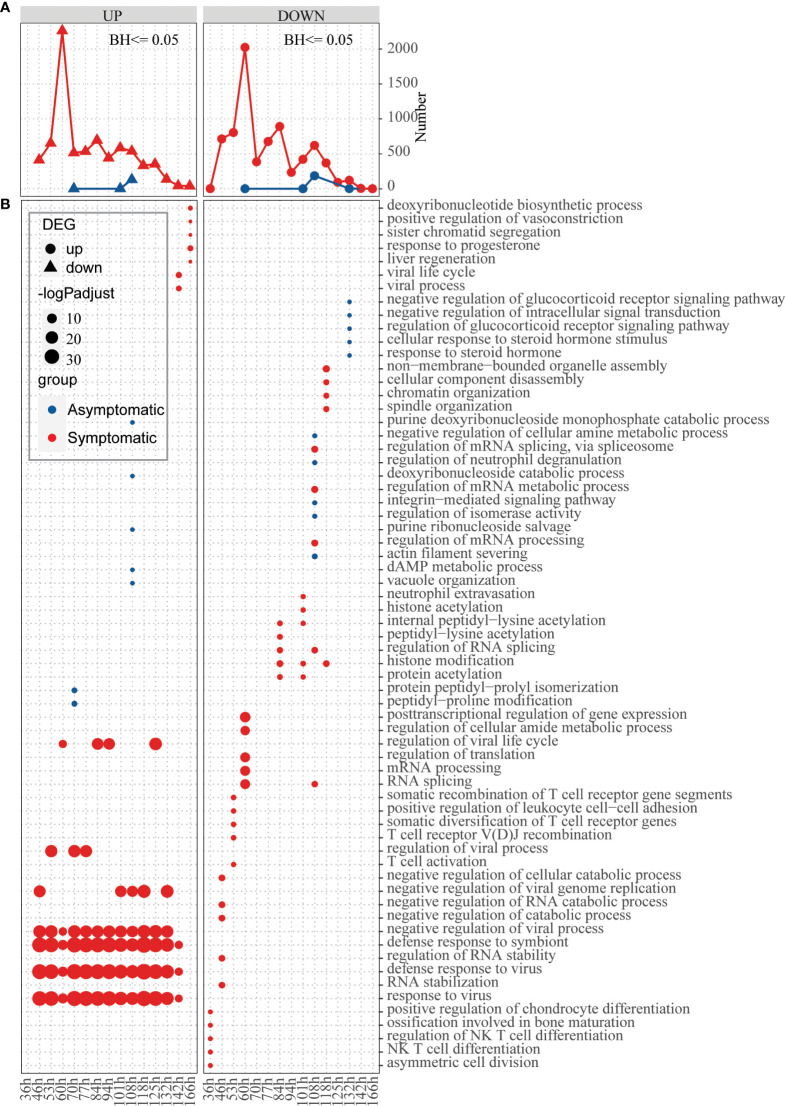
Response of hosts with different immune basis after inoculation of influenza virus. **(A)** Line chart displaying the number of up-regulated (left) and down-regulated (right) genes in the asymptomatic (blue) and symptomatic (red) hosts at different time points, respectively. **(B)** Bubble chart displaying the GO biological process terms enriched for up-regulated or down-regulated genes in the asymptomatic (blue) and symptomatic (red) hosts at different time points, respectively. Dot sizes are scaled to the enrichment significance. Different colors represent different groups.

In the above analysis, differences were observed in basic defense environment between symptomatic and asymptomatic individuals. Next, we explored how hosts with different defense basis respond to influenza invasion differently. Dataset of GSE73072-DEE2 (9 symptomatic and 8 asymptomatic hosts) that includes gene expression data at baseline and multiple time points (5h, 12h, 22h, 36h, 46h, 53h, 60h, 70h, 77h, 84h, 94h, 101h, 108h, 118h, 125h, 132h, 142h, 166h) after infection was selected for this analysis. To track the host response after infection, changes of the number of DEGs compared to baseline were extracted for each time point. Number of up-regulated and down-regulated genes firstly increased and then decreased with time in a fluctuation way, which’s highest peak at 60h in symptomatic hosts (**Figure 4A**). In the asymptomatic hosts, not only DEGs were identified at part of the time points (time points 70h,108h,118h for up-regulated genes and 60h,108h,118h, 132h for down-regulated genes), but also the number of DEGs at these time points was much less than that in the symptomatic hosts (**Figure 4A**). Overall, the symptomatic hosts had a stronger response to virus invasion than the asymptomatic hosts.

Next, function enrichment analysis for the DEGs at each time point were carried out (**Figure 4B**). Asymptomatic and symptomatic hosts induced clearly different ways of antiviral responses. For the symptomatic hosts, at the 36h, the down-regulated genes begin to significantly enrich functions, such as “asymmetric cell division”, “NK T cell differentiation”, “regulation of NK T cell differentiation” and so on. Subsequently, the up-regulated genes also began to enrich functions significantly, for example, “response to virus”, “defense response to virus”, “defense response to symbiont”, “negative regulation of viral process” and so on (**Figure 4B**, **Table S6**). But for the asymptomatic hosts, both up-regulated genes and down-regulated genes activated individual functions sporadically due to their limited number of genes. In general, symptomatic and asymptomatic individuals elicited completely different responses.

### Classification model for identifying high-risk susceptible individuals

Based on the above analyses, it seems that there are different host immune signatures for susceptibility of human seasonal influenza A/H3N2 at the baseline level. The following question is whether these baseline characteristics can help to differentiate host status of influenza susceptibility. Before building the final model, sensitivity analysis was carried out to make sure analyses here are robust. Accordingly, GSE73072 (with 22 symptomatic and 16 asymptomatic cases) were first chosen as the training set and the remaining one as the external independent test set (GSE61754, with 8 symptomatic and 3 asymptomatic cases). Based on the training dataset, the differential expression analysis (**Supplementary Figure 5**), co-expression network analysis (**Supplementary Figures 6**–**8**) and immune cell proportion analysis (**Supplementary Figures 9**, **10**) were carried out again, revealing consistent overall pattern. Then, random forest classification model was constructed based on identified significant modules (check Methods for more details), with AUC of 0.78 from the cross validation (check **Table S7** for more details). The model was also validated on the independent testing dataset with AUC of 0.875 (**Supplementary Figure 11A**, with selected genes from the modules shown in **Supplementary Figure 11B**). The final model was trained on all two data sets, with AUC of 0.78 and accuracy of 0.79 (**Table 1**, with selected genes shown in **Supplementary Figure 12**).

**Table 1:** The performance of Random Forest model to predict susceptible groups of influenza A/H3N2.

Classification Model	Accuracy	Precision	Recall	F1-score	AUC
**Random Forest**	0.79	0.81	0.87	0.83	0.78

## Discussion

In this study, attempts were carried out to reveal baseline host molecular features for susceptibility of influenza A/H3N2. Accordingly, baseline differences, especially immune conditions, between symptomatic and asymptomatic were identified based on blood transcriptomes (**Figure 1B**). The co-expression module Purple is not only positively related to asymptomatic status, but also immune cell type of naïve B cell. And further function enrichment analysis showed significantly related to “B cell receptor signaling pathway”, “immune response−activating cell surface receptor signaling pathway” and so on (**Figures 2**, **3**). Modules Lightgreen and Lightcyan are not only positively related to symptomatic status, but also immune cell type of Neutrophils, with further function enrichment analysis significantly related to “response to bacterium”, “inflammatory response”, “cAMP−dependent protein kinase complex” and so on (**Figures 2**, **3**). Further time-series investigation shows differences in response after exposure to influenza A/H3N2 between symptomatic and asymptomatic hosts, with the defense of asymptomatic hosts more effective at baseline and no need to mobilize large immune response from the view of whole blood. A classification model was also constructed with limited number of reprehensive key genes and was helpful for efficiently identifying high-risk susceptible people towards influenza A/H3N2.

Interestingly, higher co-expression modules or a higher proportion of cells associated with innate immunity were found in symptomatic hosts, but this seemingly stronger immune status did not successfully prevent the invasion of the virus (**Figures 2**, **3**). At the same time, for asymptomatic hosts, there seems a higher level for the proportion of NK cells resting (mean proportion of 1.42×10^-1^ and 1.72×10^-1^ in symptomatic and asymptomatic hosts, not statistically significant), which is consistent with previous study (16). This may be related to the double-sided role of innate immunity. For example, neutrophils as a first-line member to defend against pathogen invasion, neutrophils are undoubtedly very important (55). However, it is more and more clear recently that neutrophils contribute to the pathology of disease (56). Besides, the double-sided role of neutrophils seems to be applicable to a variety of virus-related respiratory diseases. Growing evidences have linked overactivity of neutrophils to severe disease of influenza (57), COVID-19 (58–62) and increased susceptibility of RSV (18). What’s more, neutrophils are closely related to inflammation (63–65). Inflammatory response not only prevent viral infection by preventing the replication and spread of the virus but may also cause intense lung injury and death because of overreaction (66, 67). There has been a lot of report suggested that the severity of influenza infection has a tight association with high levels of inflammation (67–70). And many anti-inflammatory drugs have been developed to treat influenza (66). This provides insights for innate immune related genes or cells or functions to be a medical target in prevention and treatment of influenza.

At the other side, the asymptomatic hosts with higher co-expression modules or a higher proportion of cells related to B cell (naïve B cell from the immune cell type analysis) may be more dominant in resisting virus invasion. Traditionally, B cells have been well known as mainly participants in adaptive immunity by differentiating into antibody-secreting cells (71), but here it is not necessarily suitable. Actually, B cells also play a key role as a regulator of innate immunity, such as B cells can regulate immune response by producing interleukin-10 (IL-10) and IL-35 (72–74). And also, except B cell related functions, the other functions, such as “defense response to virus”, “immune response−activating cell surface receptor signaling pathway”, “activation of immune response”, *etc.* were identified in **Figures 1B**, **2B, C**. Therefore, we speculate that here the whole immune state is active and ready. However, the detailed dynamics and specific mechanism needs to be further explored.

This study provides helpful insights in susceptibility of influenza A/H3N2. However, there are still several limitations and more intensive work needs to be done. Firstly, the sample size is a limitation of this work. Actually, due to ethical and security considerations, relevant data are very scarce, not to mention the detailed clinical controls (*e.g.*, age, gender, BMI, location, time, etc.). This greatly limits the study of susceptibility, and the power of analysis in this work. Even more, in the future, for better mechanistic exploration for the susceptibility of influenza and other pathogens, not only transcriptomes, but also genomes, epigenomes, proteomes, metabolomes and other omics data and high-quality clinical data need to be accumulated. Secondly, this work focuses on the transcriptome characteristics in peripheral blood at the baseline level, which is more general for clinical use. However, local mucosa such as nasal mucosa is usually the first encountering location for influenza virus invasion (75, 76), whose transcriptome characteristics at baseline and sequential times after virus invasion, roles in the susceptibility of influenza and differences from peripheral blood, need in-depth investigation. In this study, we only showed hints for susceptibility of influenza A/H3N2 from the view of whole blood and hopefully pave the way for further future investigations. Thirdly, this study focuses on one subtype of influenza (A/H3N2) and reveals characteristics of susceptibility seemingly alike to other respiratory virus-related diseases. However, there are still possible heterogeneities in virus-host interactions for hosts with different genetic/environment background and different pathogens (various subtypes of influenza and as well as various types of viruses). So the findings in our study should be careful to extrapolate to other population and other influenza subtypes or types of respiratory viruses. In the future, comprehensive comparison studies should be carried out for a variety of respiratory virus to obtain the common and specific features for the susceptibility of respiratory related viruses. Finally, further analytical and functional studies are warranted to explain the causes for the immune differences observed here (*e.g.*, different genetic backgrounds or epigenetic changes, *etc.*) and reveal the intrinsic mechanism for the susceptibility of influenza virus.

## Conclusion

In summary, potential susceptibility mechanisms were explored by comparing baseline status of blood transcriptomes between symptomatic and asymptomatic hosts in influenza challenge trials. We firstly found that there were baseline differences between symptomatic and asymptomatic hosts, especially immune related differences. Then co-expression network analysis, function enrichment analysis and immune cell proportion analysis were used to further correlate such differences with susceptible traits. In addition, based on the expression of genes from key co-expression modules, a classification model with good performance was built to identify high-risk susceptible groups for human influenza A/H3N2. These results promote the understanding of influenza susceptibility and the precise prevention and control of influenza.

## Data availability statement

The original contributions presented in the study are included in the article/**Supplementary Material**. Further inquiries can be directed to the corresponding authors.

## Author contributions

XD and WS designed the study. JT, QX, JH, YW, LY, and YL collected the data, JT, QX, KT, XY, and JZ performed the analysis. JT, ZC, MZ, and KJ built the classification model. XD, JT, WS, XG, XC, YZ, DT, and CL interpreted the data. JT prepared the manuscript. XD, WS, JT, KT, XY, XG, XC, YZ, DT, and CL edited the paper. All authors reviewed and approved the submitted manuscript.
