# Supplementary material for: Susceptibility identification for seasonal influenza A/H3N2 based on baseline blood transcriptome
Source: Front Immunol. 2023 Jan 12;13:1048774. doi: 10.3389/fimmu.2022.1048774 (PMC9878565; doi:10.3389/fimmu.2022.1048774)
Supplement: Supplementary file 8 [file DataSheet_8.docx]

Supplementary material

**Susceptibility identification for seasonal influenza A/H3N2 based on baseline blood transcriptome**

# Data Availability Statement

The source code and related data are available on GitHub (<https://github.com/DuLab-SYSU/FluSusceptibility>).

# Supplementary Figures and Tables

**Table S1 Influenza challenge experiments**

**Table S2 Results of GO enrichment related to Figure 1B**

**Table S3 Genes in the co-expression module**

**Table S4 Results of GO enrichment related to Figure 2B**

**Table S5 Results of KEGG enrichment related to Figure 2C**

**Table S6 Results of GO enrichment related to Figure 4B**

**Table S7 The performance of Random Forest model to predict susceptible groups of influenza A/H3N2 on the selected training dataset**

# Supplementary Figures


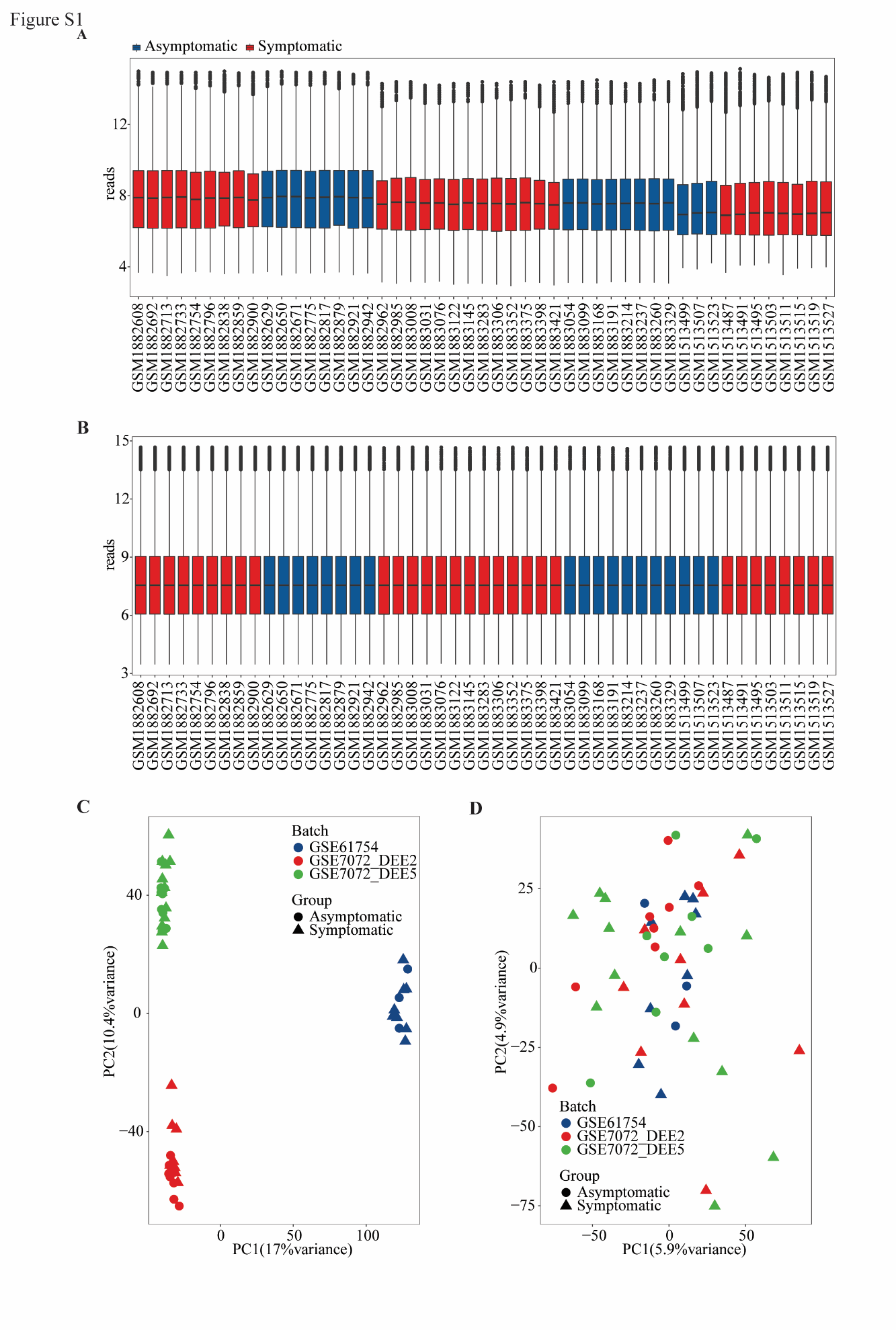


**Supplementary Figure 1 Data Pre-procession**

**A**. Intensity boxplots before normalization. The color represents the type of host (red: Symptomatic; blue: Asymptomatic).

**B**. Intensity boxplots after normalization. The meaning of colors is same with Supplementary Figure 1-A.

**C** and **D**. The principal component analysis (PCA) for genes in the microarrays we collected before (left) and after (right) the batch effects remove. Different colors represent the influenza challenge experiments of the data belong to, and also represent different batches. Different shapes represent the group of samples.


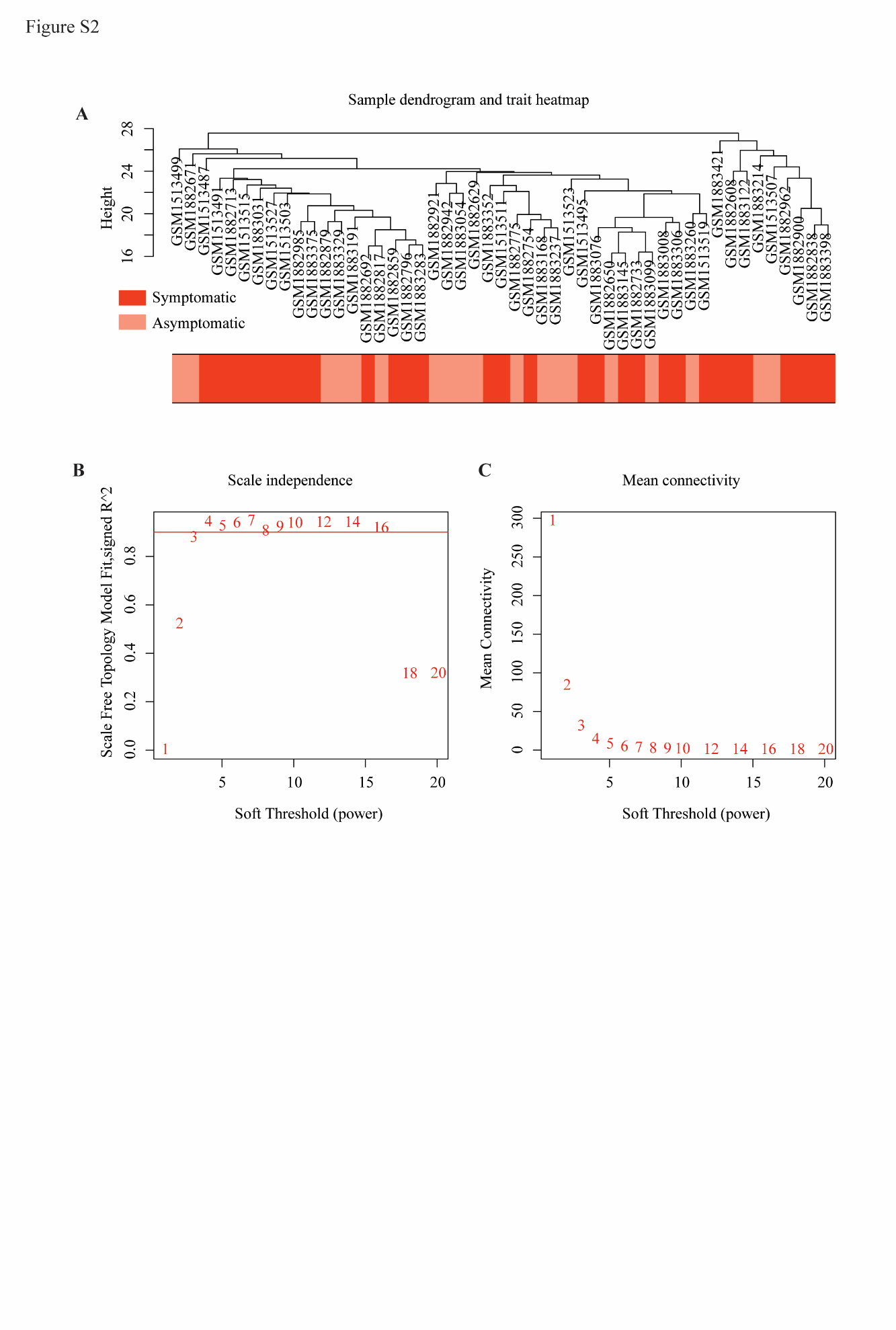


**Supplementary Figure 2 Co-expression network construction**

**A**. Clustering dendrogram of 49 samples.

**B**. Analysis of the scale-free fit index (y-axis) for various soft thresholding powers (x-axis).

**C**. Analysis of the mean connectivity (degree, y-axis) for various soft thresholding powers (x-axis).


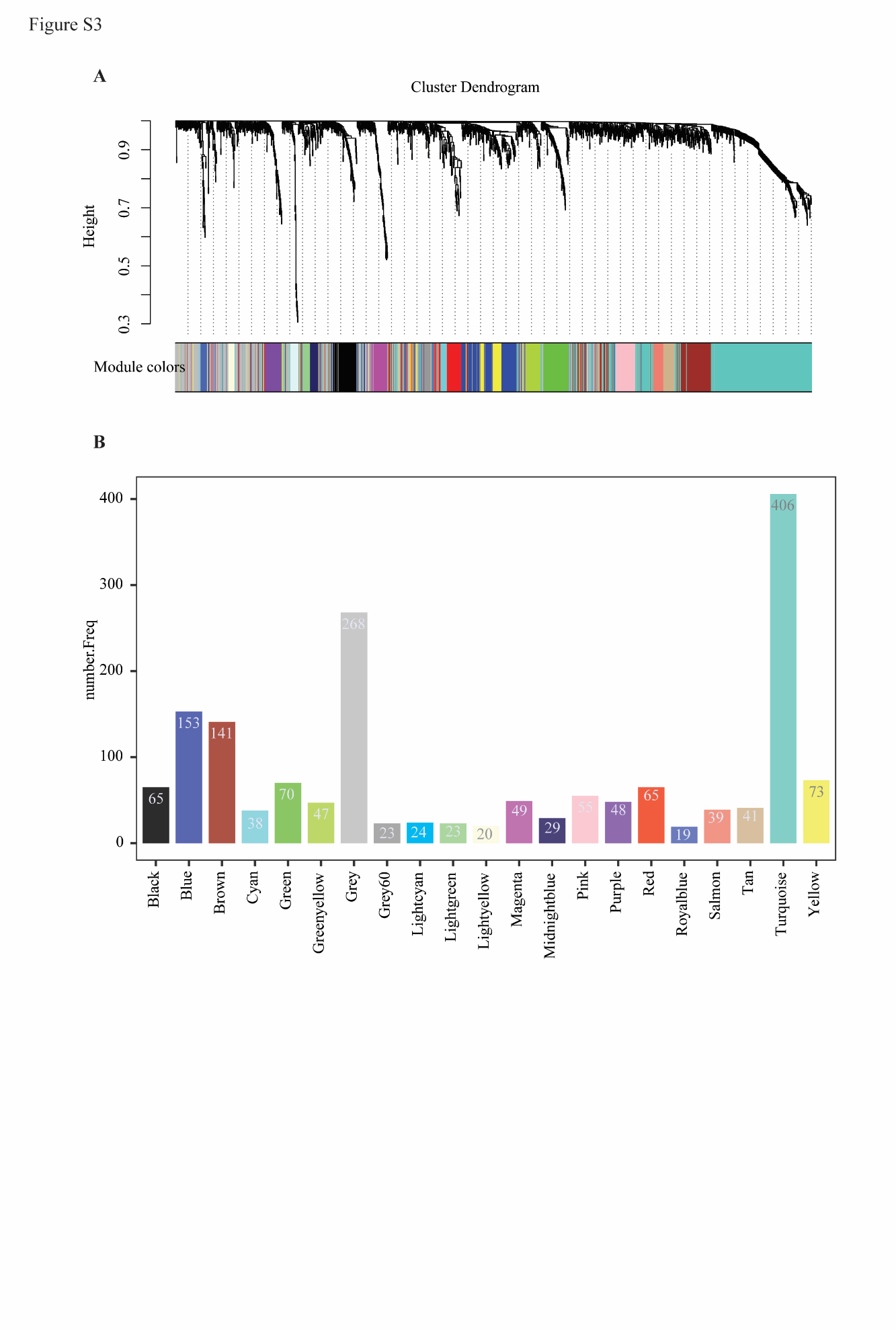


**Supplementary Figure 3 Properties of co-expression network**

**A.** Dendrogram of genes in the top 20% of variance clustered by hierarchical clustering. There were 18 types of modules, representing by different colors.

**B.** Bar plots showing the number of genes in the 18 modules. Different colors represent different modules.


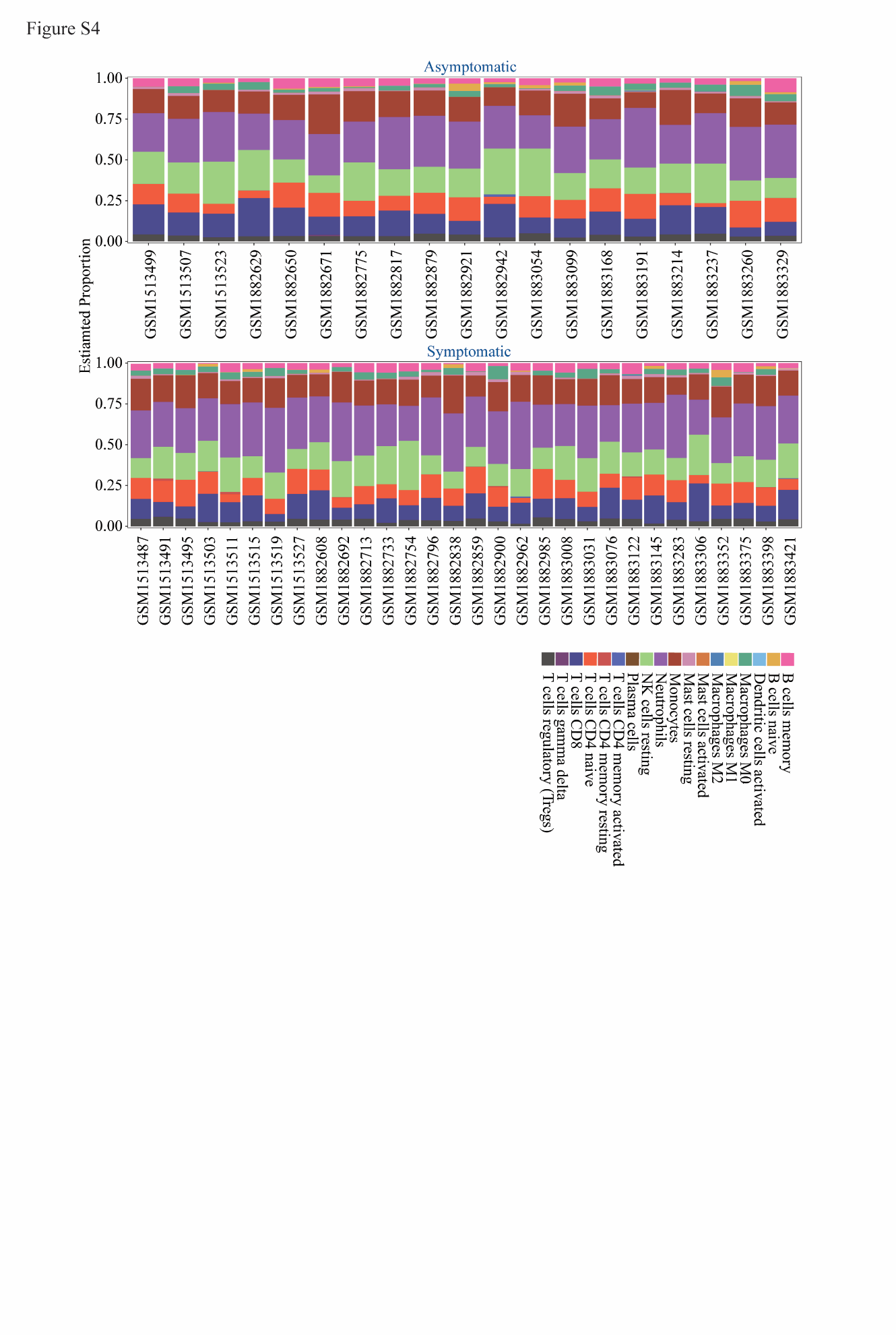


**Supplementary Figure 4. Immune cell proportions**

Cell type proportions estimated by CIBERSORT for each sample in the whole dataset. Different colors represent different immune cell types.


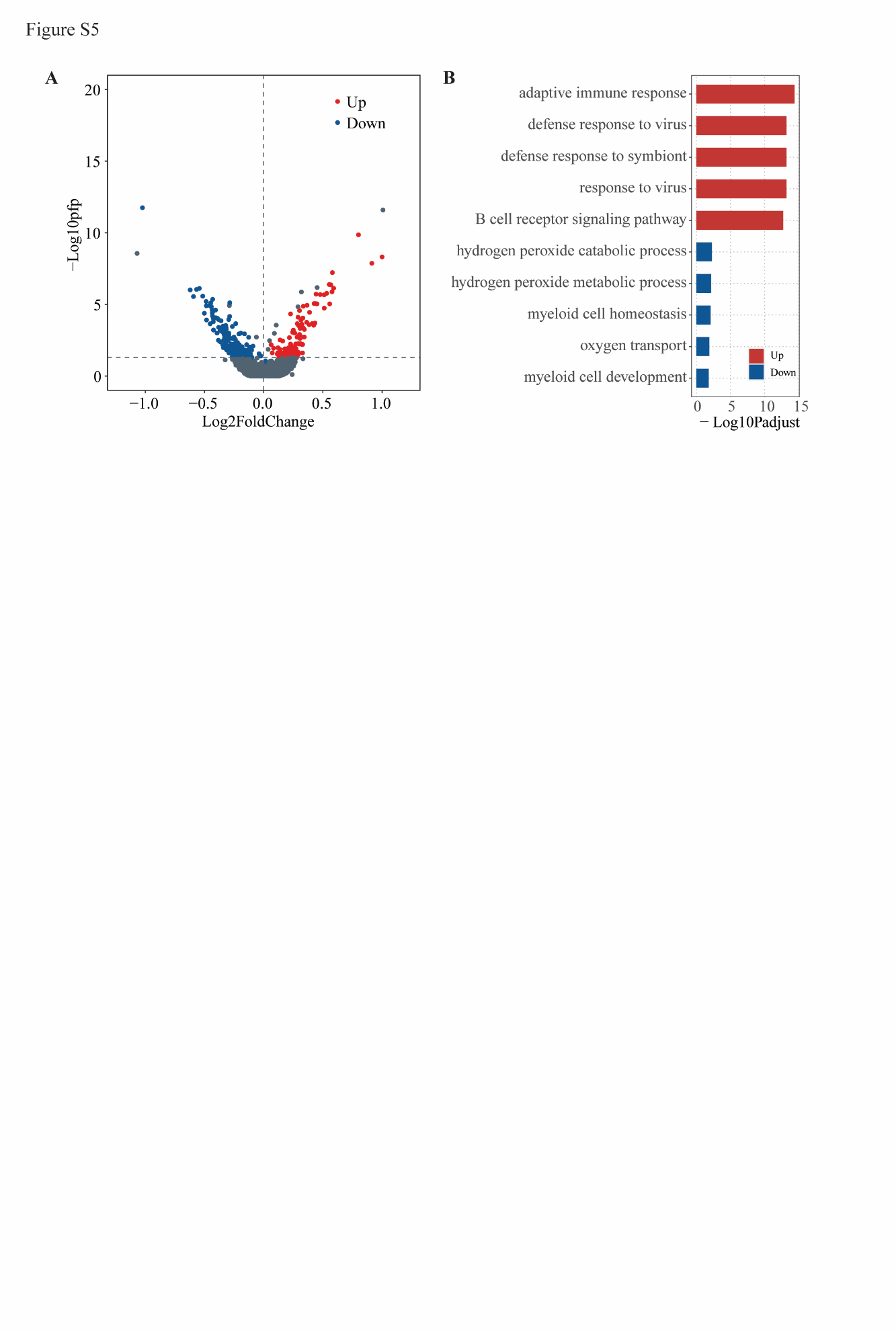


**Supplementary Figure 5 DEGs at baseline level between asymptomatic and symptomatic hosts based on the selected training datasets**

**A.** Volcano plot showing logarithmically converted fold change of gene expression between asymptomatic and symptomatic hosts on the x-axis against logarithmically converted values of *pfp* on the y-axis. The red points are up-regulated genes, and the blue points are down-regulated genes and the grey points are stable genes (asymptomatic vs symptomatic hosts).

**B.** Bar plot showing the GO biological process terms enriched for the up- (red) and down- (blue) regulated genes. The x-axis represents the logarithmically transformed *Padjust* value.


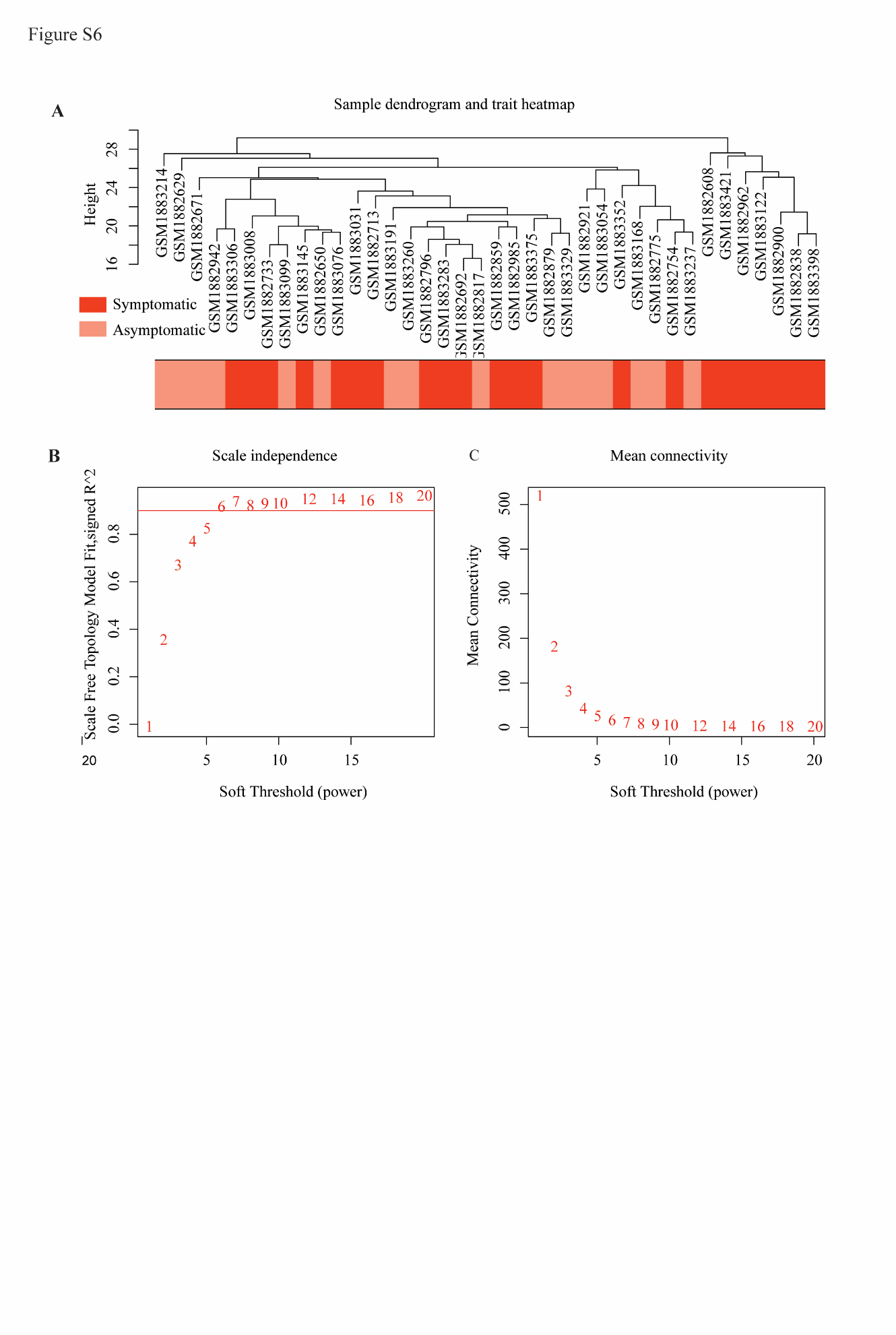


**Supplementary Figure 6 Co-expression network construction using the selected training datasets**

**A.** Clustering dendrogram of 38 samples.

**B.** Analysis of the scale-free fit index (y-axis) for various soft thresholding powers (x-axis).

**C**. Analysis of the mean connectivity (degree, y-axis) for various soft thresholding powers (x-axis).


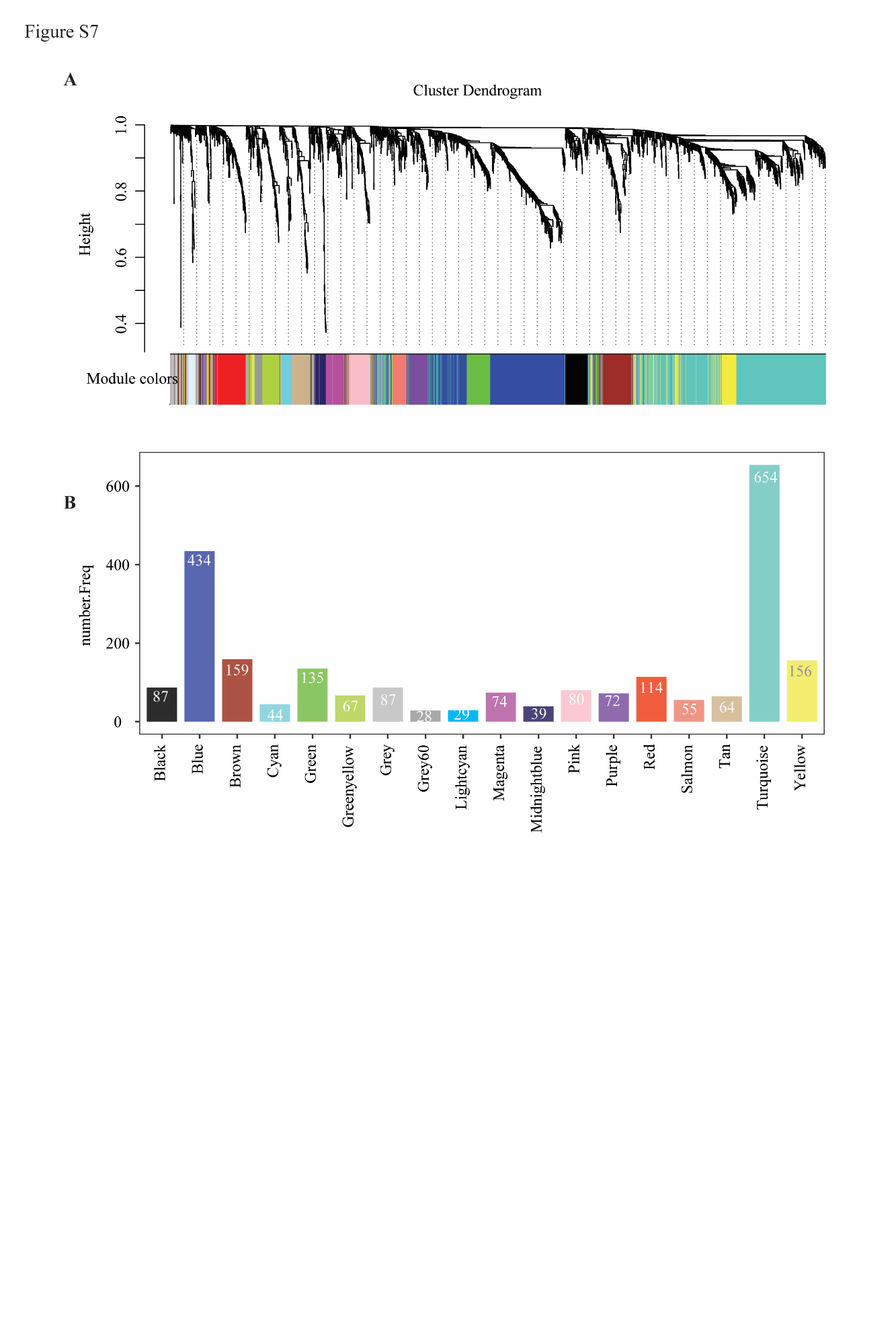


**Supplementary Figure 7 Properties of Co- expression network using the selected training datasets**

**A.** Dendrogram of genes in the top 20% of variance clustered by hierarchical clustering. There were 18 types of modules, representing by different colors.

**B.** Bar plots showing the number of genes in the 18 modules. Different colors represent different modules.


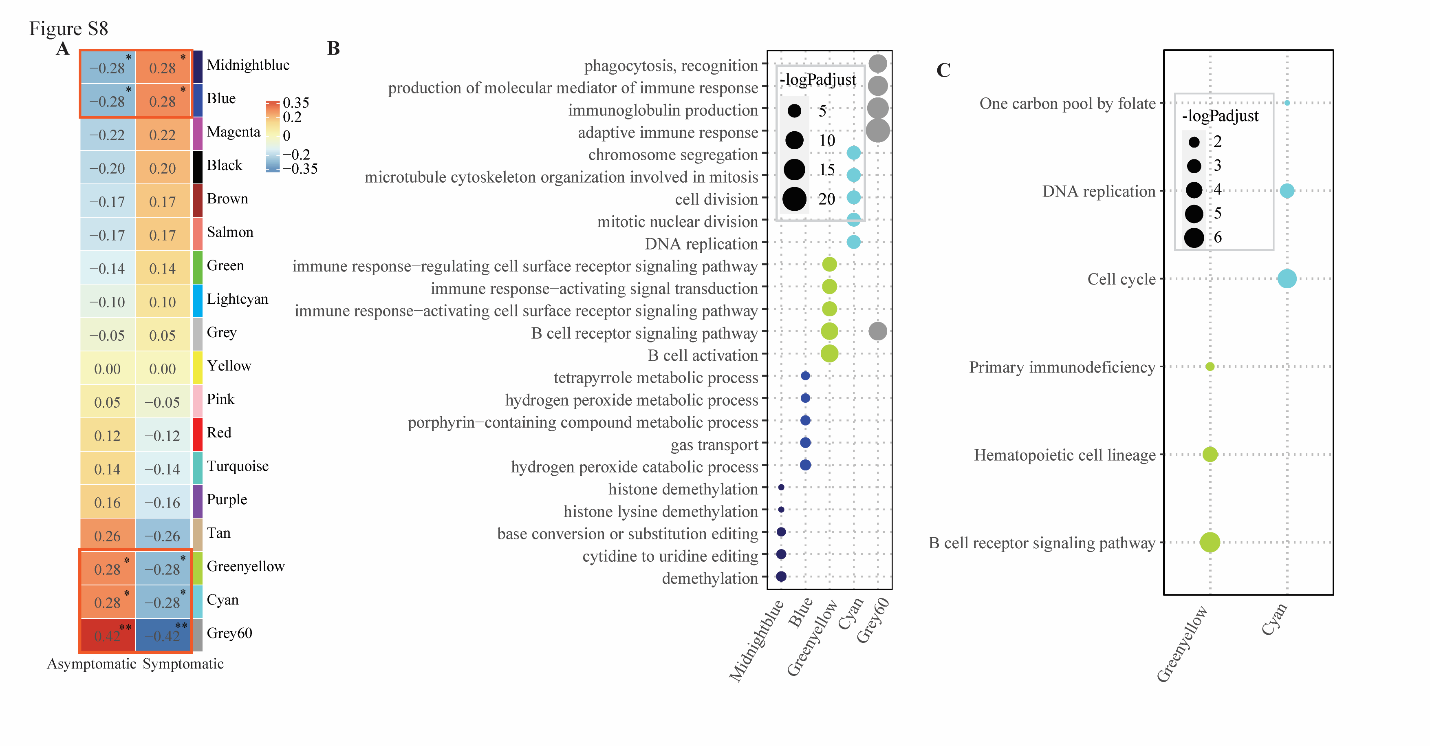


**Supplementary Figure 8 Identification of core modules of the co-expression network based on the selected training datasets**

**A.** Heatmap of Module-trait’s correlation, in which traits on the x-axis against the co-expression modules on the y-axis. The corresponding correlation was shown in the cells and color coded. The significantly related modules were indicated by number of asterisks (* for p ≤ 0.1, ** for p ≤ 0.01, and *** for p ≤ 0.001) and highlighted by red rectangles.

**B.** Bubble chart displaying the GO terms enriched for the five significant modules (Midnightblue, Blue, Greenyellow, Cyan, Grey60). Dot sizes are scaled to the enrichment significance. Different colors represent different modules.

**C.** Bubble chart displaying the KEGG pathways enriched for the three significant modules (Midnightblue, Blue, Greenyellow, Cyan, Grey60). Dot sizes are scaled to the enrichment significance. Different colors represent different modules.


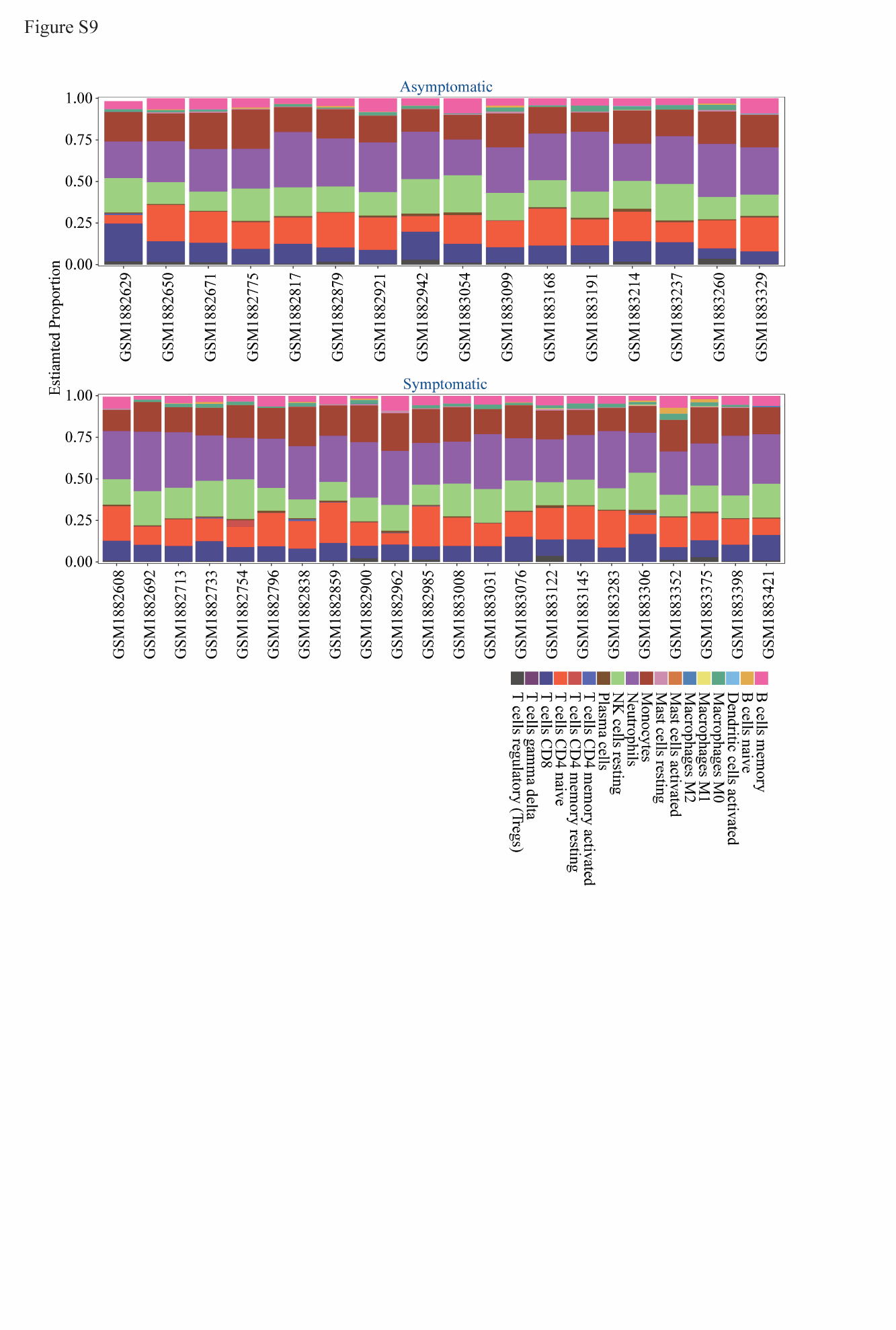


**Supplementary Figure 9. Sample’s immune cell proportions in the selected training datasets**

Cell type proportions estimated by CIBERSORT for each sample contained in the training dataset. Different colors represent different immune cell types.


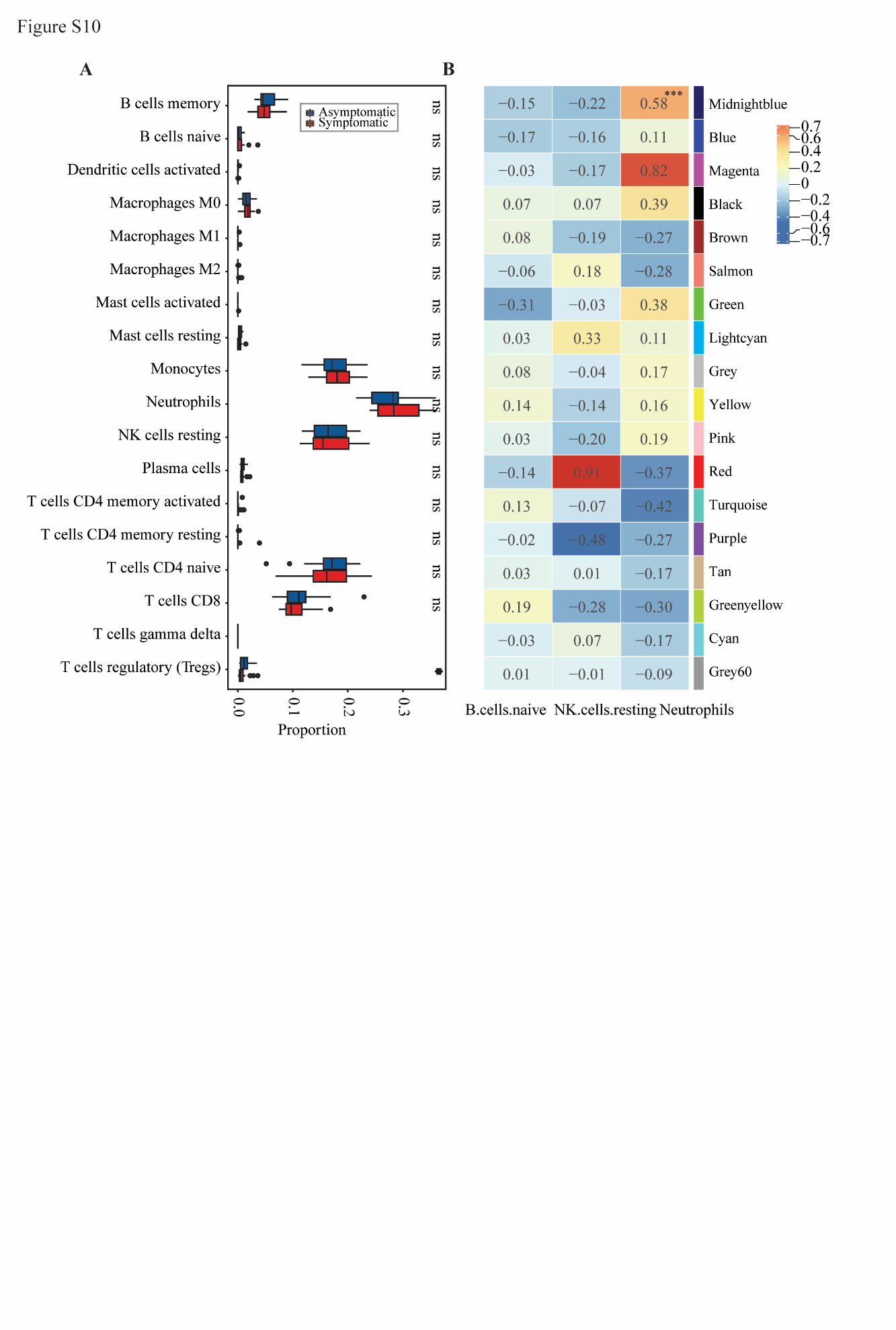


**Supplementary Figure 10 Immune cell proportions’ differences in the selected training dataset and their relationships with co-expression modules.**

**A.** Boxplot showing the immune cell proportions in asymptomatic (blue) and symptomatic (red) hosts. *Wilcoxon rank-sum test* was used to determine significant differences between asymptomatic and symptomatic hosts. The significant level was indicated by number of asterisks (ns for p >0.1, * for p ≤0.1, ** for p ≤ 0.01, and *** for p ≤ 0.001) and highlighted by red rectangle.

**B.** Heatmap of Module-trait’s correlation, in which immune cell types on the x-axis against the co-expression modules on the y-axis. The corresponding correlation was shown in the cells and color coded. The significantly related modules were indicated by number of asterisks (* for p ≤ 0.1, ** for p ≤ 0.01, and *** for p ≤ 0.001) and highlighted by red rectangles.


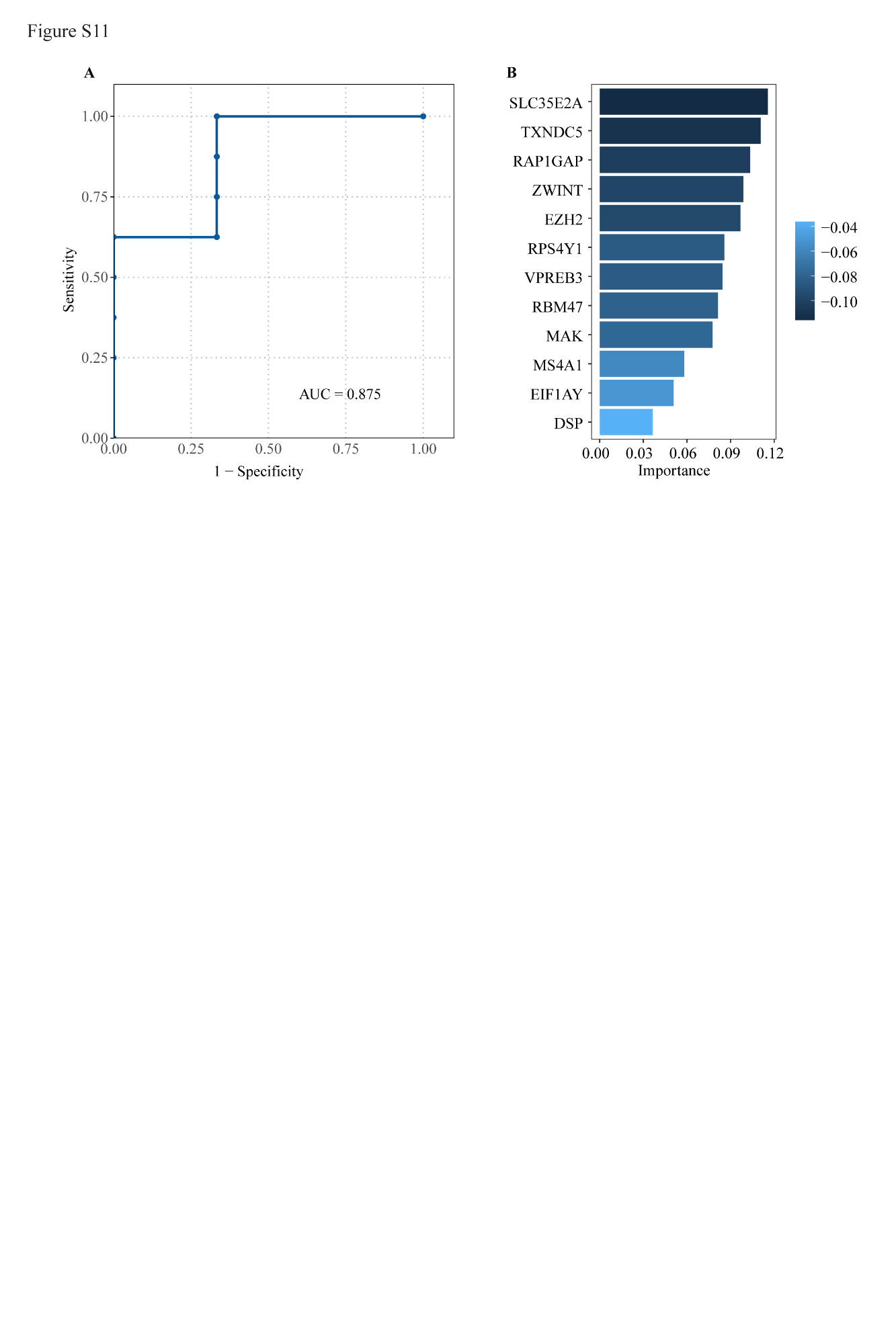


**Supplementary Figure 11 Performance of** **random forest classification model**

**A.** ROC curve of Random Forest model for identifying symptomatic hosts based on the test dataset. The x-axis ‘1-sensitivity’ is the proportion of symptomatic hosts that are detected. The y-axis ‘specificity’ is the proportion of asymptomatic hosts that are detected.

**B.** Bar plot showing the importance of genes in the classification model based on the selected training dataset. The x-axis represents the importance. The color corresponds the value of importance.


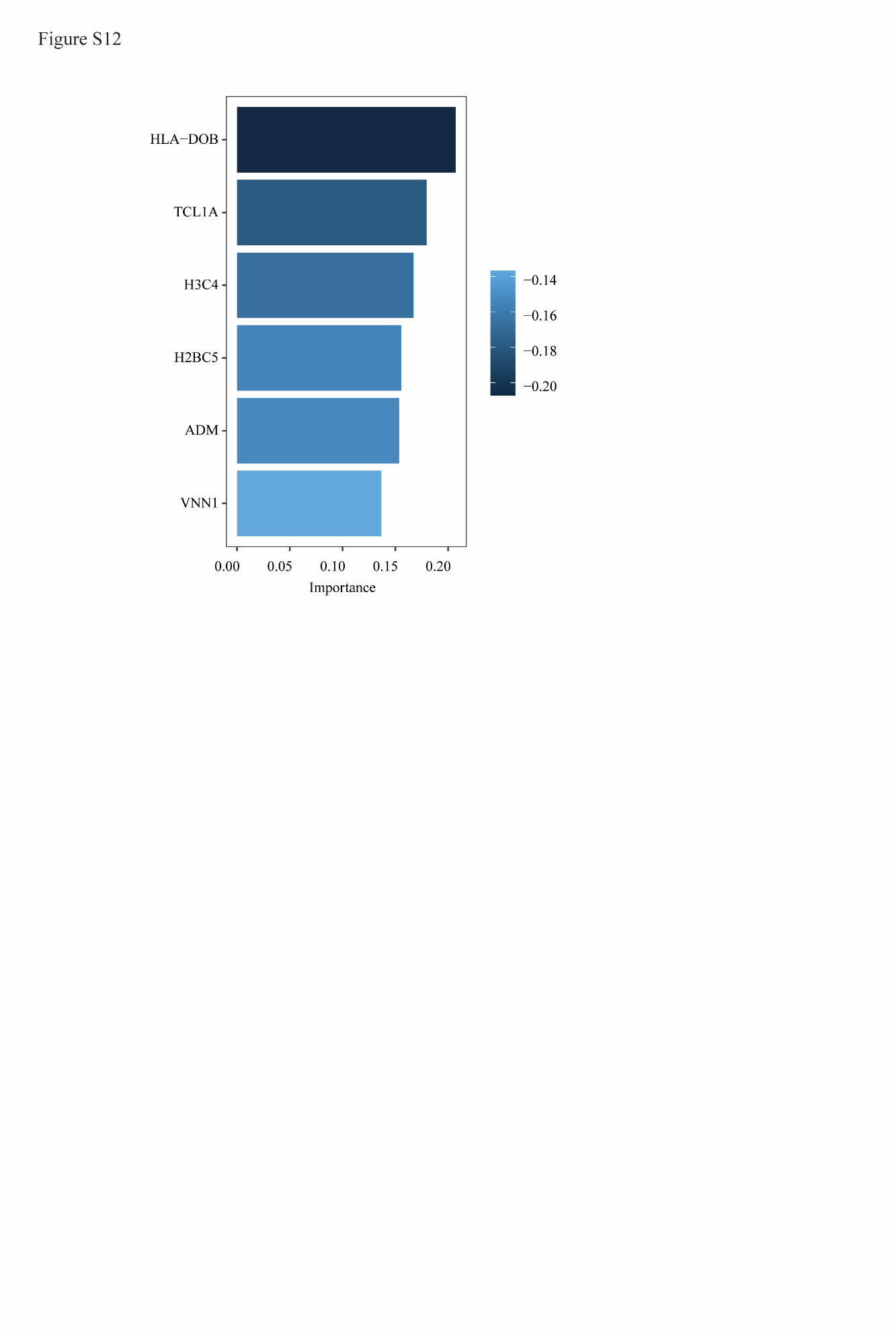


**Supplementary Figure 12 Feature importance**

Bar plot showing the importance of genes in the classification model based on the whole dataset, respectively. The x-axis represents the importance. The color corresponds the value of importance.
